# Antithrombotic mechanism of polysaccharides in Blackberry (*Rubus* spp.) seeds

**DOI:** 10.1080/16546628.2017.1379862

**Published:** 2017-10-03

**Authors:** Jinmei Wang, Pengli Lian, Qi Yu, Jinfeng Wei, Wenyi Kang

**Affiliations:** ^a^ Institute of Chinese Materia Medica, Henan University, Kaifeng China; ^b^ Kaifeng Key Laboratory of functional components in health food, Kaifeng China

**Keywords:** Blackberry seed, polysaccharides, structural, anticoagulant, antithrombotic

## Abstract

The blackberry seed was typically removed as a byproduct and waste from blackberry fruits for juices. Developing value-added utilization of berry seeds will significantly expand the market for berry products as well as improve benefit to berry producers. However, the research on blackberry seed is limited. The objective of this paper was to research antithrombotic mechanism of polysaccharides in blackberry seeds. Polysaccharides in blackberry seeds were extracted, purified and identified by high-performance gel permeation chromatography (HPSEC), gas chromatography (GC), fourier transform infrared (FT-IR) spectrometer and nuclear magnetic resonance spectra (NMR). Anticoagulant activities were evaluated *in vivo* by measuring activated partial thromboplastin time (APTT), thrombin time (TT), prothrombin time (PT), fibrinogen (FIB) and plasma recalcification time (RRT). Four polysaccharides named BSP-1a, BSP-1b, BSP-2 and BSP-3 were isolated from Blackberry (*Rubus* spp.) seeds. The results indicated that BSP-1b, BSP-2 and BSP-3 exhibited the anticoagulant activity. Therefore, the anti-thrombosis effects of BSP-1b, BSP-2 and BSP-3 were investigated *in vivo* by 6-Keto-PGF_1α_, thromboxane B_2_ (TXB_2_), endothelial nitric oxide synthase (eNOS), endothelin-1 (ET-1), whole blood viscosity (WBV), plasma viscosity (PV), hematocrit (Hct), erythrocyte sedimentation rate (ESR), APTT, TT, PT and FIB. The results suggested that BSP-1b, BSP-2 and BSP-3 had the inhibition effect on thrombus formation, and the antithrombotic effects were associated with the regulation of vascular endothelium active substance, activating blood flow and anticoagulation effect.

## Introduction

Blackberry (*Rubus* spp.) is a widely distributed shrub with high commercial value due to its sensory and chemical characteristics,such as sugars, malic acid, amino acids, VC, VE, anthocyanins, superoxide dismutase (SOD) and flavonoids [1]. Therefore, it is usually used as raw material for the production of jams, candy, wine and juice beverages, and so on. The seeds of the blackberry fruits were typically removed during juice processing. In our previous research, 13 compounds were isolated and identified from blackberry seeds, pharmacology research indicated that blackberry seeds had antioxidant [2], cholesterol-lowering [3], liver-protection effects [4], and could rectify the derangement in lipide metabolism [5]. However, there is no research on polysaccharides from blackberry seeds in literature.

Plant polysaccharides display a wide variety of biological activity, including immunostimulatory [6], anti-cancer [7], anti-diabetic [8] and antithrombotic activities [9]. As already shown for algae polysaccharides, so do plant polysaccharides have anticoagulant activity [10–12], and can be used as a prevention and treatment of cardiovascular disease [13]. The aim of this study was to isolate, characterize and evaluate the anticoagulant and antithrombotic effects of the polysaccharides of blackberry seeds. The results may provide a reference for the further research and development of Blackberry (*Rubus* spp.).

## Materials and methods

### Plant materials

Seeds of Blackberry (*Rubus* spp.) were collected in July 2013 from the blackberry planting base of Fengqiu (Henan, China) and were identified by Professor Chang-qin Li in the Department of Pharmacognosy, Henan University. The voucher specimens were deposited at Traditional Chinese Medicine Research Institute of Henan University.

### Animals

Male rabbit (2.0–2.5 kg), were provided by traditional Chinese medicine research institute of Henan University.

Female and male Sprague-Dawley (SD) rats (200–250 g) were purchased from Laboratory Animal Center, Zhengzhou, Henan, China. They were maintained under a 12/12°h light/dark cycle, at 25 ± 2°, with free access of food and water.

### Chemicals

DEAE-cellulose-52 was purchased from Whatman; Sephadex G-100 was purchased from Pharmacia; Kits for activated partial APTT, PT, TT and FIB were from Shanghai Sun Biotech Co., Ltd; Rat 6 Keto-PGF_1α_ ELSA kit, rat TXB_2_ ELSA kit, Rat ET-1 ELSA kit and Rat eNOS ELSA kit were from Nanjing senbeijia biotech Co., Ltd.

### Extraction and purification of polysaccharide

The dried Blackberry seeds (200 g) were ground into powder and defatted three times by petroleum ether at room temperature. The organic solvent was volatilized to obtain a dry powder. Then the powder was extracted three times with 70% ethanol and filtered. Finally, the residue was lyophilized. Subsequently, the dried powder was extracted with 20 volumes of distilled water at 80° every 3 h for three times. The aqueous extract was filtered and the supernatant was treated with 95% ethanol (final concentration 70%) at 4° overnight, and centrifuged at 10,000 rpm for 10 min. The precipitation was added with Sevage reagent (chloroform/1-butanol, 1:4 v/v) for deproteinization deproteinisation [14]. The crude polysaccharide was obtained through precipitation with 95% ethanol (final concentration 70%) and centrifuged. Then the precipitate was redissolved in water and dialyzed for 2 days to remove small molecular weight impurity (molecular weight cut off 3500 Da). Finally, the aqueous extract was lyophilized in vacuum to give the crude polysaccharide (9.7 g).

The 300 mg crude polysaccharides were dissolved in 10 mL distilled water, and then the water was filtered through 0.45 μm microporous membrane. Then the filtered water was fractioned by DEAE-52 column (2.5 × 60 cm). The column was eluted with distilled water at 0.8 mL·min^−1^ followed by 0.1 M NaCl and 0.2 M NaCl, respectively. The fractions were collected using an automated step-by-step fraction collector and guided for total carbohydrate using the phenol-sulfuric acid method [15]. Three main fractions were collected, dialyzed, lyophilized and named as BP-1(74 mg), BP-2 (104 mg)and BP-3(95 mg). These polysaccharides were further purified through a column of Sephadex G-100 (1.5 × 100 cm) and eluted with water at 0.5 mL·min^−1^. The purified fraction was combined, concentrated and lyophilized for further study.

Moreover, four polysaccharides were analysed for concents of total carbohydrates, protein and uronic acid using phenol-sulfuric acid, bradford [16] and m-hydroxydiphenyl [17].

### Molecular weight analysis

The molecular weight of polysaccharides was identified by high-performance size-exclusion chromatography [18]in Beijing centre for physical and chemical analysis.

### Monosaccharide composition analysis

Polysaccharide samples (10 mg) were hydrolyzed in ampoules with 2 M trifluoroacetic acid (2 mL) for 3 h at 110°, evaporated and added with methanol to remove TFA. Then the hydrolyzates were mixed with 10 mg hydroxylamine hydrochloride and 0.5 mL pyridine and incubated at 90° for 30 min. Acetic anhydride (0.5 mL) was added and incubated at 90°for 30 min [19]. The mixtures were cooled to room temperature, and filtered through 0.22 μm filters. The resulting alditol acetates were analysed by gas chromatograph (GC), which was performed on a Thermo TRACE1300 instrument (Milan, Italy) fitted with FID (280°) and equipped with TG-Waxms column (30 m × 0.32 mm×0.5 μm). The column temperature was maintained at 100° for 1 min, and increased to 230° for 10 min at a rate of 4°·min^−1^ with N_2_ as the carrier gas. The standard monosaccharides were prepared and subjected to GC analysis separately in the same way.

### Spectroscopic analysis

The infrared spectrum of four polysaccharides were measured on a FT-IR spectrometer (Vertex 70, Bruker, Germany) using KBr pellets in the infrared region of 4000 − 500 cm^−1. 1^H NMR analyses of them were carried out on an Avance Bruker III HD 400 MHz NMR spectrometer.

### Anticoagulation assays *in vitro*

#### Anticoagulation time assay

Anticoagulation activities of APTT, PT, TT and FIB were performed *in vitro* and the assay was conducted by using rabbit blood collected from rabbit ear veins in plastic tubes containing 3.8% sodium citrate (citrate/blood: 1/9, v/v). Then, the blood was centrifuged at 3000 rpm for 15 min at 5°C to obtain the serums. For APTT assay, 25 μL of sample solution tested was mixed with 50 μL of citrated normal rabbit serum, and then APTT assay reagent was added. Following, the mixture was incubated at 37°C for 5 min. Then 25 mM CaCl_2_ solution (100 μL) was added into the incubated mixture to initiate the reaction. Finally, the clotting time was recorded. For PT assay, samples (25 μL) were mixed with serum (25 μL) and incubated at 37°C for 3 min. While PT assay reagent (50 μL), which had been hatched for 10 min at 37°C, was then added and clotting time was recorded. TT and FIB assays were performed according to the manufacturer’s specifications [20]. For all clotting assays, blank solvent was used as the blank control group, and breviscapine and Vitamin K_1_ were used as the positive control group, and the time for clot formation was recorded by a Semi-Automated Coagulation Analyser (Han Fang, Jinan, China).

### PRT assays *in vitro*

Blood samples were collected from rabbit ear veins in the plastic tubes containing 3.8% sodium citrate (citrate/blood: 1/9, v/v). Then, the blood was centrifuged at 1000 rpm for 15 min to obtain the plasma. Tested samples (100 μL) were mixed with 100 μL of citrated normal rabbit plasma, and then RPT assay reagent was added. All experimental groups were adding 0.1 mL of CaCl_2_ solution (0.925 mg/mL) after training 1 min at 37°. The control group, coagulant control and anti-coagulant control were repeated three times, the sample group was repeated six times [21].

### Anti-thrombosis activity assays *in vivo*

#### Experimental model

The SD rats were randomly divided into seven groups with six animals in each. The groups included the blank control group, model group, positive group and three-treated group. The rats of the blank control group and model group were given the corresponding blank solvent (distilled water) by gavage. The positive control group was given aspirin (100 mg/kg). The three treated groups were given BSP-1b (120 mg/kg), BSP-2 (120 mg/kg) and BSP-3 (120 mg/kg), respectively. All of the groups were given by gavage twice a day for 7 days.

After the fifth administration, blood stasis model was established, except for the blank control rats. The model rats were injected with adrenaline hydrochloride (0.8 mg/kg). After 2 h of the first administration of epinephrine hydrochloride subcutaneously, the rats were placed in ice water (from 0 to 2°) for 5 min, and then injected with adrenaline hydrochloride (0.8 mg/kg) again 2 h after the ice-bath. Subsequently, rats were fasted for 12 h with free access to water and administration continued.

The rats were anesthetized with 10% chloral hydrate (300 mg/kg) 30 min after the last administration. Then the blood samples were collected from abdominal aorta to determine the corresponding parameters.

### Plasma coagulation parameters *in vivo*

Blood samples were collected from abdominal aorta into the plastic tubes containing 3.8% sodium citrate (citrate/blood: 1/9, v/v). Then, the blood was centrifuged at 1000 rpm for 15 min to obtain the plasma. The determination method for APTT, PT, TT and FIB accorded with anticoagulation assays *in vitro.*

### Determination of TXB2, 6-keto-PGF1α, eNOS and ET-1

The preparation method of the plasma was reference to plasma coagulation parameters *in vivo*. The plasmas of TXB_2_, 6-keto-PGF_1α_, eNOS and ET-1 were performed by enzyme-linked immunoassay according to the manufacturer’s recommended procedures.

### Hemorheology parameters

The WBV and PV of the samples were measured by a Auto-Viscometer. For WBV, the blood samples mixed with heparin sodium were placed into the sample cell of the Auto-Viscometer. Then, WBV was measured with shear rates of 200/s, 20/s and 3/s at 37°C. For PV, the blood was centrifuged at 1000 rpm for 15 min to obtain the plasma, and then PV was measured by the viscometer. Hct was determined by the Wintrobe method and ESR was determined by Westergren method.

### Statistical analysis

All experimental results were expressed as mean ± standard deviation (SD). Statistical analysis was performed with the SPSS19.0 software. Comparison between any two groups was evaluated using one-way analysis of variance (ANOVA).

## Results and discussion

### Extraction and purification of polysaccharide

Crude polysaccharides were successfully isolated by a series of experimental procedures such as water extraction, deproteination, dialysis, ethanol precipitation and lyophilization. The crude polysaccharides were then separated by using DEAE-cellulose-52 column and further purified by Sephadex G-100. As a result, four purified fractions were obtained, named as BSP-1a(34 mg), BSP-1b(21 mg), BSP-2(50 mg) and BSP-3 (46 mg). BSP-1a contained 96.31% of total carbohydrate and 1.58% of protein. BSP-1b contained 94.29% of total carbohydrate and 2.71% of protein. BSP-2 contained 93.76% of total carbohydrate, 4.06% of protein and 8.91% of uronic acid. BSP-3 contained 92.35% of total carbohydrate, 2.12% of protein and 7.22% of uronic acid.

### Molecular weight analysis

Molecular weight of polysaccharide was a statistical average, which was representative of similar polymer chain length distributed on average. The average molecular weight (*Mw*) of the four polysaccharides was 16,868, 178,500, 33,600 and 224,300 g/mol, respectively.

### GC analysis

The monosaccharide composition of BSP-1a was galactose, fructose, rhamnose, arabinose and xylose in a molar ratio of 1.42:1.48:1.35:19.59:7.83. BSP-1b was composed of galactose, fructose, rhamnose, arabinose and xylose with a molar ratio of 19.13:2.27:3.30:5.48:1.78. The monosaccharide composition of BSP-2 was determined to be galactose, fructose, rhamnose, arabinose and xylose in a ratio of 2.55:1.95:7.40:17.98:8.71. BSP-3 was composed of galactose, fructose, rhamnose and arabinose in a ratio of 0.58:0.91:3.41:2.63.

### FT-IR

**Figure 1.**
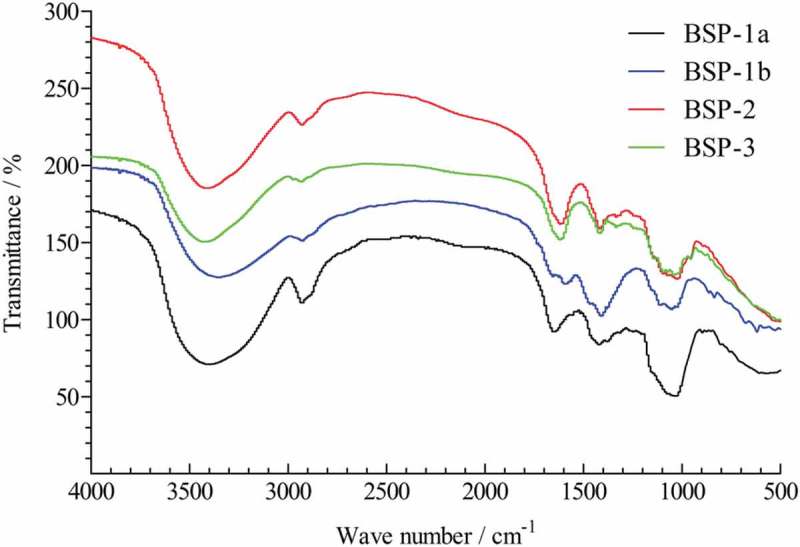
Infrared spectra of BSP-1a, BSP-1b, BSP-2 and BSP-3.

The FT-IR spectroscopy of BSP-1a, BSP-1b, BSP-2 and BSP-3 were scanned between 4000 and 500 cm^−1^ and the results showed the four polysaccharides were similar to each other (Figure 1). The absorption band was found in all samples between 3351 cm^−1^ and 3417 cm^−1^, indicating the presence of hydroxyl group. The appearance of the peaks within the range of 2800–2950 cm^−1^ was due to the presence of the C-H stretching vibration. The signals at around 1597–1654 cm^−1^ and 1409–1420 were showing the presence of carboxyl groups. Absorption at 1010–1100 the C-O and C-C stretching vibrations of pyranose ring. Moreover, the characteristic absorption between 820 cm^−1^ and 890 cm^−1^ indicated that *α*-glycosidic linkages and *β*-glycosidic linkages in the polysaccharides.

### ^1^H NMR spectral analysis

**Figure 2.**
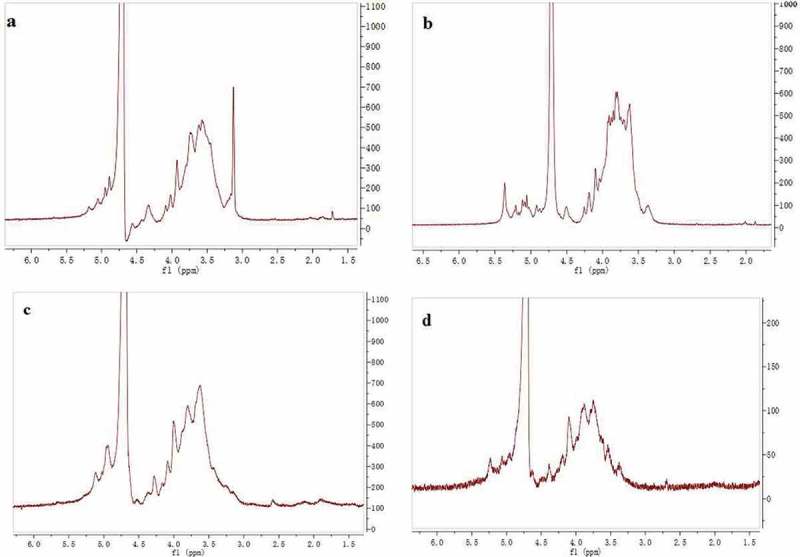
^1^H NMR spectrum of BSP-1a (a), BSP-1b (b), BSP-2 (c) and BSP-3 (d).

As shown in Figure 2, the anomeric region of the ^1^H NMR spectrum showed mostly of signals at 4.0–5.5 ppm for the four polysaccharides. The chemical shifts from *δ* 5.0 to 5.5 ppm were attributed to α-glycosidic linkage, and the signals between *δ* 4.0 and 5.0 ppm were assigned to *β*-glycosidic linkage. These findings were in accordance with FT-IR spectrum.

### Anticoagulation assays *in vitro*

The anticoagulant assays of BSP-1a, BSP-1b, BSP-2 and BSP-3 were tested by PT, APTT, TT, FIB and PRT *in vitro*. PT is used to evaluate the coagulation factors V, VII and X in the overall efficiency of extrinsic clotting pathway. On the other hand, APTT is a test of the coagulation factors VIII, IX, XI, XII and Von Willebrand’s factor [22] in the intrinsic clotting activity. TT is mainly a measure of transformation of fibrinogen to fibrin degree. FIB is employed to reflect the content of fibrinogen. The PRT is an important indicator for measuring the activity of the endogenous coagulation process [23]

In Table 1, compared with the blank group, BSP-1a could significantly shorten APTT, PT and TT (*P *< 0.001) and could significantly increase the content of FIB (*P *< 0.001), which indicated that BSP-1a had coagulant activities and exerted the coagulant activities through intrinsic pathway, extrinsic pathway and increased the content of FIB. Compared with the blank group, BSP-1b could significantly prolong PRT, APTT, TT and PT (0.01 < *P *< 0.05, or *P *< 0.001, respectively), so the anticoagulant activities of BSP-1b was associated with the intrinsic pathway and extrinsic pathway. Compared with the blank group, BSP-2 could significantly prolong PRT, APTT and TT (*P *< 0.001, respectively) and could significantly decrease the content of FIB (*P *< 0.001), but PT had no difference with the blank group (*P *> 0.05), and thus suggested that BSP-2 had anticoagulant activities and exerted the anticoagulant activities through intrinsic pathway and increased the content of FIB. So the anticoagulant activities of BSP-2 were associated with the intrinsic pathway and extrinsic pathway. Compared with the blank group, BSP-3 could significantly prolong PRT, APTT, TT and PT (0.01 < *P *< 0.05, or, *P *< 0.001, respectively), and could significantly decrease the content of FIB (*P *< 0.001), which indicated that BSP-3 had anticoagulant activities and exerted the anticoagulant activities through intrinsic pathway, extrinsic pathway and decreased the content of FIB.

**Table 1.** Anticoagulation activity of BSP-1a, BSP-1b, BSP-2 and BSP-3.

Group	PRT(s)	APTT(S)	PT(S)	TT(s)	FIB(g/L)
blank	219.7 ± 3.2	20.50 ± 0.08	9.93 ± 0.10	32.78 ± 0.10	3.38 ± 0.02
breviscapine	274.5 ± 5.2^###^	32.73 ± 0.13^###^	10.95 ± 0.06^###^	39.55 ± 0.13^###^	5.29 ± 0.17^###^
vitamin k1	164.1 ± 2.0^###^	13.53 ± 0.13^###^	7.55 ± 0.06^###^	19.67 ± 0.17^###^	—
BPS-1a	237.7 ± 3.7^###ΔΔΔ^	15.68 ± 0.13^###ΔΔΔ^	8.63 ± 0.15^###ΔΔΔ^	31.25 ± 0.06^###ΔΔΔ^	3.83 ± 0.12^###^***
BPS-1b	342.0 ± 4.9^###^***	45.70 ± 0.08^###^***	10.45 ± 0.42^##^**	53.20 ± 1.40^###^***	3.41 ± 0.09^#^***
BPS-2	318.8 ± 3.8^###^***	24.15 ± 0.06^###^***	9.90 ± 0.14^#^	39.65 ± 1.43^###^*	3.12 ± 0.14^###^***
BPS-3	352.8 ± 8.8^###^***	28.30 ± 0.08^###^***	10.83 ± 0.15^###^*	61.50 ± 3.05^###^***	2.65 ± 0.10^###^***

Compared with Blank (0.01  <  *P < 0.05, 0.001<**P < 0.01, *** *P *< 0.001);

Compared with Breviscapine (0.01 <^#^ *P *< 0.05, 0.001<^##^ *P *< 0.01, ^###^ *P *< 0.001).

Compared with Vitamin k1 (0.01 <  ^Δ^ *P *< 0.05, 0.001 <  ^ΔΔ^ *P *< 0.01, ^ΔΔΔ^ *P *< 0.001).

### Anti-thrombosis activity assays *in vivo*

TXB_2_ is a degradation product of thromboxane A_2_ (TXA_2_) and 6-keto-PGF_1α_ is a metabolite of epoprostenol (PGI_2_). TXA_2_ and PGI_2_ are extremely unstable in the body. Thus, to detect the contents of TXB_2_ and 6-keto-PGF_1α_ can reflect TXA_2_ and PGI_2_ levels. Research evidence has indicated that TXA_2_ is a platelet activating agent, which can promote the formation of thrombus. PGI_2_ is a platelet function inhibitor, which has a protective effect on the vasospasm caused by platelet aggregation. The balance between biosynthesis of PGI_2_ and that of TXA_2_ is important in the prevention of thrombosis [24–27].

**Figure 3.**
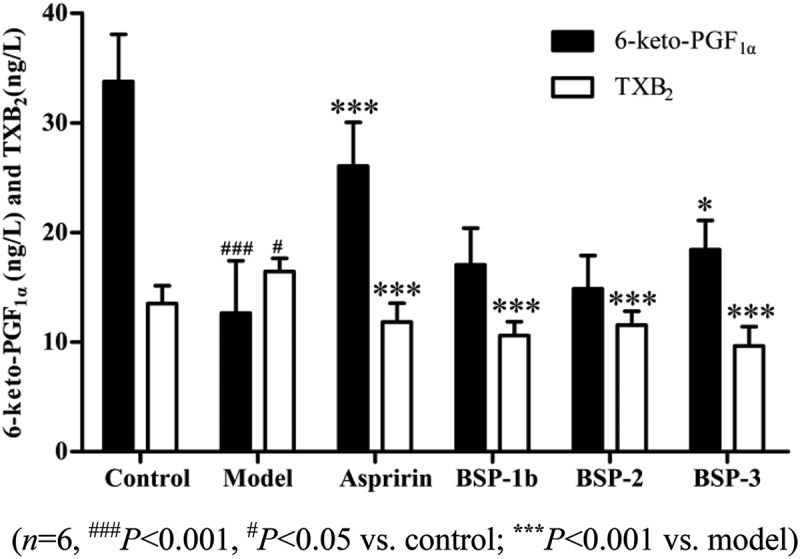
Effect on 6-keto-PGE_1α_ and TXB2 in rats. (*n *= 6, ^###^ *P *< 0.001, ^#^ *P *< 0.05 vs. control; *** *P *< 0.001 vs. model).

As shown in Figure 3, compared with the model group, BSP-1b, BSP-2 and BSP-3 could significantly increase the content of 6-Keto-PGF_1α_ and reduce the content of TXB_2_ (*P *< 0.001), indicating that the antithrombotic mechanism of BSP-1b, BSP-2 and BSP-3 was associated with the balance of TXB_2_ and 6-keto-PGF_1α_.

ET-1, which is widely found in vascular endothelium, plays an important role in cardiovascular function. Vascular contraction, myocardial ischemia, metabolic disorder and cell proliferation induced by ET-1 are the common pathogenic factors of vascular injury. The eNOS can be activated to generate endogenous NO, which can inhibit the formation of thrombosis through regulating the tension of the vascular smooth, inhibiting platelet aggregation and vascular smooth muscle cell proliferation.

**Figure 4.**
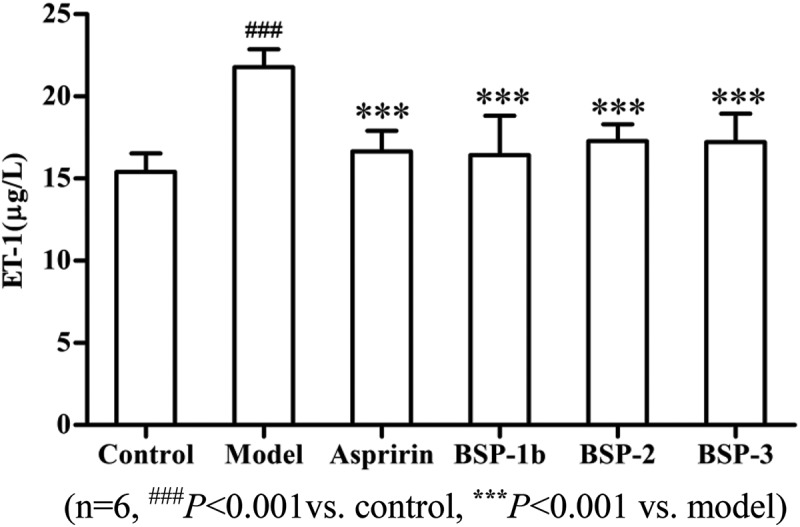
Effect on ET-1 in rats. (n = 6, ^###^ *P *< 0.001vs. control, *** *P *< 0.001 vs. model).

**Figure 5.**
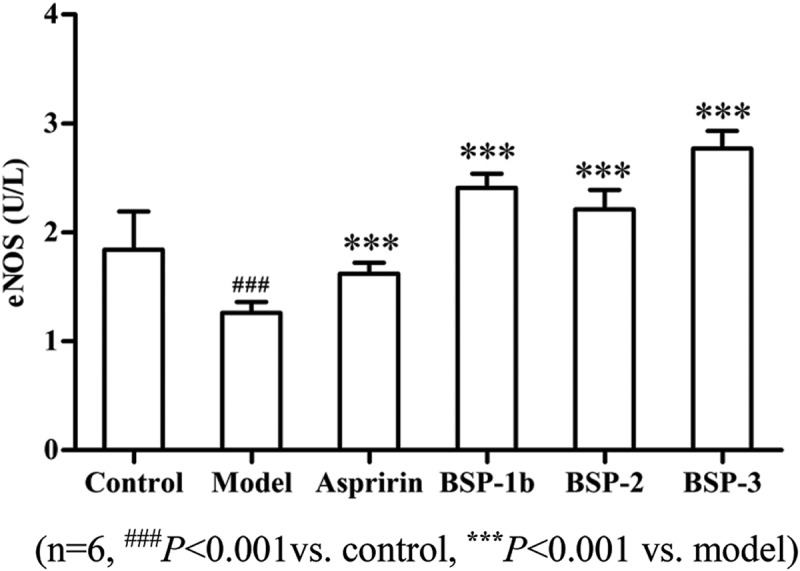
Effect on eNOS in rats. (n = 6, ^###^ *P *< 0.001vs. control, *** *P *< 0.001 vs. model).

In Figures 4 and 5, compared with the model group, BSP-1b, BSP-2 and BSP-3 could significantly reduce the content of ET-1 and could significantly increase the content of eNOS (*P *< 0.001). The result indicated that BSP-1b, BSP-2 and BSP-3 could inhibit the formation of thrombosis by regulating the content of ET-1 and eNOS [28].

WBV reflects the intrinsic resistance of hemokinesis in blood vessels and it varies according to the different shear rate. The abnormality of WBV can increase the resistance of blood fluidity and slow down the blood flow velocity, thus leading to the cardiovascular risk. Hct is the most important factor for WBV [29–32]. In Table 2, compared with model group, BSP-1b, BSP-2 and BSP-3 could significantly decrease WBV at all shear rates (0.001 < *p *< 0.05, *p *< 0.001), as well as BSP-1b, BSP-2 and BSP-3 could significantly decrease Hct (*p *< 0.001). So BSP-1b, BSP-2 and BSP-3 could reduce the WBV by decreasing Hct. Moreover, PV and ESR play an important role in WBV. The results of ESR and PV are shown in Table 3. Compared with the model group, BSP-1b, BSP-2 and BSP-3 could significantly decrease ESR and PV (P < 0.001, or, 0.001 < P < 0.01, or, P < 0.05), suggested that the amelioration effect of administered groups on WBV might be the decrease of ESR and PV.

**Table 2.** Effect on WBV and Hct.

		WBV(mPa·s)			
Group	Dose (mg/kg)	200/s	20/s	3/s	Hct(%)
Blank	–	3.54 ± 0.31	4.60 ± 0.29	9.23 ± 0.13	37.85 ± 0.93
Model	–	4.28 ± 0.23^###^	5.94 ± 0.35^###^	11.11 ± 0.35^###^	41.96 ± 0.91^###^
Aspirin	100	3.83 ± 0.37^Δ^	4.78 ± 0.46^ΔΔΔ^	9.53 ± 0.19^ΔΔΔ^	39.62 ± 0.78^ΔΔ^
BSP-1b	120	3.80 ± 0.32^Δ^	5.12 ± 0.14^ΔΔΔ^	9.94 ± 0.44^ΔΔΔ^	39.58 ± 1.36^ΔΔ^
BSP-2	120	3.89 ± 0.20^Δ^	5.08 ± 0.15^ΔΔΔ^	10.34 ± 0.60^Δ*^	39.42 ± 1.56^ΔΔ^
BSP-3	120	3.91 ± 0.29^Δ^	5.30 ± 0.24^ΔΔ^	10.24 ± 0.79^Δ**^	40.12 ± 0.62^Δ^

Data represent mean ± SD. *n *= 6;

Compared with blank (^###^ *P *< 0.001)

Compared with model (^ΔΔΔ^ *P *< 0.001, 0.01 < ^ΔΔ^ *P*<0.01, ^Δ^ *P * 0.05)

Compared with aspirin (0.001 < ** *P *< 0.01, * *P *< 0.05)

**Table 3.** Effect on ESR and PV.

Group	Dose (mg/kg)	ESR (mm/h)	PV (mPa·s)
Blank	–	3 ± 0.82	1.36 ± 0.06
Model	–	11.5 ± 1.29^###^	1.53 ± 0.09^##^
aspirin	100	7 ± 0.82^ΔΔΔ^	1.39 ± 0.04^ΔΔ^
BSP-1b	120	4.25 ± 0.95^ΔΔΔ**^	1.38 ± 0.07^ΔΔ^
BSP-2	120	7.25 ± 1.7^ΔΔΔ^	1.36 ± 0.09^ΔΔ^
BSP-3	120	8.5 ± 1.3^ΔΔΔ^	1.43 ± 0.05^Δ^

Data represent mean ± SD. n = 6;

Compared with blank (^###^ *P *< 0.001, 0.001<^##^ *P *< 0.01)

Compared with model (^ΔΔΔ^ *P *< 0.001, 0.01 < ^ΔΔ^ *P *< 0.01, ^Δ^ *P*< 0.05)

Compared with aspirin (0.001 < ** *P *< 0.01)

In Table 4, compared with the model group, BSP-2 and BSP-3 could significantly prolong APTT, PT and TT, and could significantly reduce the content of FIB (P < 0.001), suggesting BSP-2 and BSP-3 had the antithrombotic, and the activities were associated with the intrinsic pathway and extrinsic pathway, and decreased the content of FIB. BSP-1b could significantly prolong APTT, PT and TT (P < 0.001), suggesting BSP-1b could inhibit the formation of antithrombotic and the activities were associated with the intrinsic pathway and extrinsic pathway.

**Table 4.** Coagulation parameters of BSP-1a, BSP-1b, BSP-2 and BSP-3.

		Plasma coagulation parameters			
Group	Dose (mg/kg)	APTT(s)	PT(s)	TT(s)	FIB(g/L)
blank	–	44.45 ± 1.87	23.4 ± 0.53	36.05 ± 1.37	2.3 ± 0.12
model	–	23.88 ± 1.21^###^	18.62 ± 0.55^###^	18.58 ± 0.62^###^	3.9 ± 0.22^###^
aspirin	100	34.47 ± 0.63^ΔΔΔ^	20.5 ± 0.52^ΔΔΔ^	32.8 ± 1.11^ΔΔΔ^	3.44 ± 0.13^ΔΔΔ^
BSP-1b	120	39.85 ± 1.89^ΔΔΔ***^	24.53 ± 0.39^ΔΔΔ***^	28.25 ± 0.99^ΔΔΔ^	3.72 ± 0.14
BSP-2	120	37.19 ± 1.21^ΔΔΔ*^	26.48 ± 0.92^ΔΔΔ***^	29.35 ± 1.06^ΔΔΔ^	3.22 ± 0.23^ΔΔΔ^
BSP-3	120	38.93 ± 0.83^ΔΔΔ***^	27.38 ± 0.79^ΔΔΔ***^	29.4 ± 0.48^ΔΔΔ^	2.83 ± 0.11^ΔΔΔ***^

Data represent mean ± SD. n = 6;

Compare with Blank (^###^ *P *< 0.001, 0.001<^#^ *P *< 0.05)

Compare with model (^ΔΔΔ^ *P *< 0.001)

Compare with aspirin (*** *P *< 0.001, * *P *< 0.05)

## Conlusion

In our study, the crude polysaccharides were obtained from the Blackberry seeds. Sevage reagent and dialysis bag were used to remove proteins and small molecular weight impurity, respectively. Then, BSP-1a, BSP-1b, BSP-2 and BSP-3 were obtained by anion exchange chromatography and sephadex column chromatography. The results of anticoagulant activity of *in vitro* indicated that BSP-1b, BSP-2 and BSP-3exhibited the anticoagulant activity. Therefore, the anti-thrombosis activity assays of BSP-1b, BSP-2 and BSP-3 were taken for further study. The results suggested that BSP-1b, BSP-2 and BSP-3 had the inhibition effect on thrombus formation in blood stasis model rats, and the antithrombotic activities were associated with the regulation of vascular endothelium active substance, activating blood flow and anticoagulation activity. These findings provided a pharmacological basis for application in clinical of BSP-1b, BSP-2 and BSP-3 on blood stasis syndrome. In addition, these results could be used to create a new industrial field for the production of fish farm supplements, as well as other nutraceuticals [33–36].
